# A review on the diversity, phylogeography and population genetics of *Amanita* mushrooms

**DOI:** 10.1080/21501203.2015.1042536

**Published:** 2015-06-09

**Authors:** Ping Zhang, Li-Ping Tang, Qing Cai, Jian-Ping Xu

**Affiliations:** a College of Life Science, Hunan Normal University, Changsha410081, China; b School of Pharmaceutical Sciences and Yunnan Key Laboratory of Pharmacology for Natural Products, Kunming Medical University, Kunming650500, China; c Key Laboratory for Plant Diversity and Biogeography of East Asia, Kunming Institute of Botany, Chinese Academy of Sciences, Kunming650201, China; d Department of Biology, McMaster University, Hamilton, ONL8S 4K1, Canada

**Keywords:** *Amanita*, ectomycorrhizal fungi, phylogeny, molecular markers

## Abstract

*Amanita* mushrooms are important for both human beings and ecosystems. Some members in this genus are valued edible species, whereas some others are extremely poisonous, and most species are ectomycorrhizal. Significant progress has been made in recent years in our understanding of the diversity, phylogeography and population genetics of *Amanita* mushrooms. A significant reason for the progress was due to the increasing application of molecular methods in the analyses. In this review, we summarize the researches in the diversity, phylogeography and population genetics of *Amanita* mushrooms, with the focus on advances over the past 20 years. We also discussed future research directions, including several unresolved topical issues.

## Introduction

*Amanita* Pers. is one of the most specious and best-known fungal genera. The genus comprises about 500 described species and likely a similar number of undescribed species (Bas 2000; Yang 2000a; Tulloss 2005). Because it contains both deadly poisonous species, e.g. *Amanitaphalloides* (Vaill. ex Fr.) Link and famous edible species, e.g. *Amanitacaesarea* (Scop.) Pers., this genus has attracted the attention of mycologists since the very beginning of scientific mycology (Persoon 1801; Fries 1821). Moreover, a large majority of the species in this genus form ectomycorrhizal (EM) relationships with vascular plants and play important roles in ecosystems (Yang 1997). With the introduction of molecular methods at the end of last century in analysing the natural history of this genus (Weiß et al. 1998; Drehmel et al. 1999), our knowledge of genus *Amanita* has increased rapidly. The aim of this review is to summarize the progress about the diversity, phylogeography and population genetics of amanitas, emphasizing the results from the last 20 years.

## Diversity

**Figure 1.**
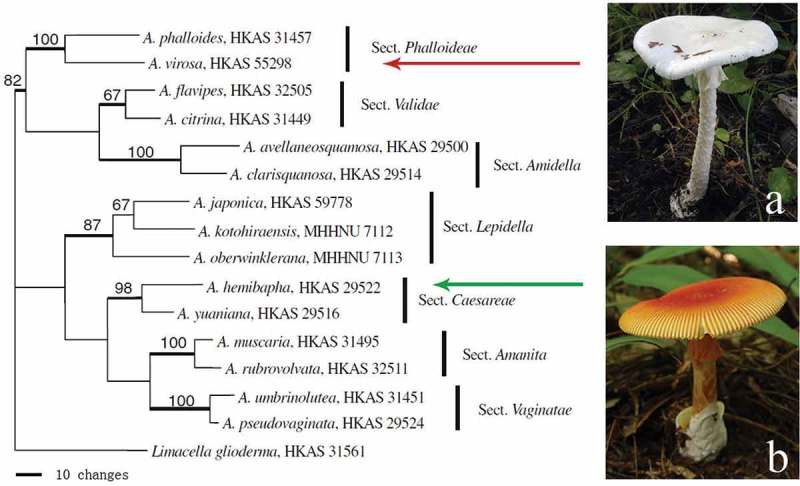
Phylogenetic position of a lethal species, *A. virosa* (a), and an edible species, *A. hemibapha* (b), in a most parsimonious tree of genus *Amanita* based on nuclear large subunit (nLSU) sequences (Zhang et al. 2010).

*Amanita* mushrooms belong to Basidiomycota, Agaricomycetes, Agaricales and Amanitaceae. They are characterized by having (usually) white, free to subfree gills with bilateral lamellar trama, white spore print, volval remants as warts or patches on the pileal surface and the base of the stipe (Yang and Oberwinkler 1999). In addition, many have an annulus on the stem. This genus is divided into seven sections: *Amanita, Caesareae* Singer, *Vaginatae* (Fr.) Quél., *Amidella* (J.-E. Gilbert) Konrad & Maubl., *Lepidella* (J.-E. Gilbert) Veselý, *Phalloideae* (Fr.) Quél., and *Validae* (Fr.) Quél (Yang 1997). Most of the lethal species are included in section *Phalloideae*, whereas most of the edible species belong to the section *Caesareae* (Figure 1).

### New taxa

It has been estimated that there are 900–1000 species of *Amanita* worldwide (Tulloss 2005). Of these, about half have been described. Among these described species, about 100 are considered poisonous and about 50 are edible. For the remaining species, their edibility is largely unknown. Over the last two decades, about 220 new taxa (new species, new varieties and new forms) in *Amanita* have been reported from all over the world, especially in East Asia, Central and South America, South Africa and Australia. While many of these were due to the analyses of new samples from previously under-sampled geographic regions, the application of molecular markers helped reveal a significant number of new taxa (cryptic species) among existing collections, similar to those found in many other groups of basidiomycetes (Yang 2011).

Here, because of the large number of new taxa, we will not describe all the new species in detail. Instead, we will provide a representative summary of new species from diverse geographic regions. For example, Oda et al. (2001, 2002a, 2002b, 2002c) reported five species of *Amanita* from Japan. Interestingly, among these five species, *Amanitaareolata* was later found to be a synonym of *Amanitazangii*, and *Amanitagriseoturcosa* was later transferred from the section *Phalloideae* to the section *Lepidella* (Cai et al. 2014). Nagasawa and Mitani (2000) also reported a new species in the section *Lepidella*. Based on the intensive studies on amanitas from China and adjacent areas, Yang (2005) published Flora Fungorum Sinicorum Vol. Amanitaceae, and described 26 new species (Yang and Doi 1999; Yang et al. 1999, 2001, 2004; Yang 2000a, 2000b, 2002; Yang and Li 2001; Yang and Zhang 2002). However, despite the comprehensive update, additional species were continuously described from China. For example, Zhang et al. (2010) reported three lethal amanitas in East Asia. Deng et al. (2014) and Li and Cai (2014) each described a new *Amanita* species from South China. In other parts of Asia, many new specie were also found. For example, five new taxa were found in India (Bhatt et al. 2003) and Pakistan (Tulloss et al. 2001).

Outside of Asia, Simmons et al. (2002) reported four new species of *Amanita* from Guyana. Tulloss et al. (1992) studied the amanitas from Andean Colombia, and described 11 new species (or new varieties). Eicker et al. (1993) reported a new species named *Amanita reidii* from South Africa. However, because *A. reidii* was associated with *Eucalyptus*, he considered it an introduced species from Australia. Wood (1997) did extensive studies on genus *Amanita* in Australia and reported 34 new species. Even in Europe and North America, where *Amanita* had been intensively studied by fungal taxonomists, new *Amanita* taxa have also reported (Tulloss and Lindgren 1994; Tulloss et al. 1995; Neville and Poumarat 2004).

### Infraspecific variations

**Figure 2.**
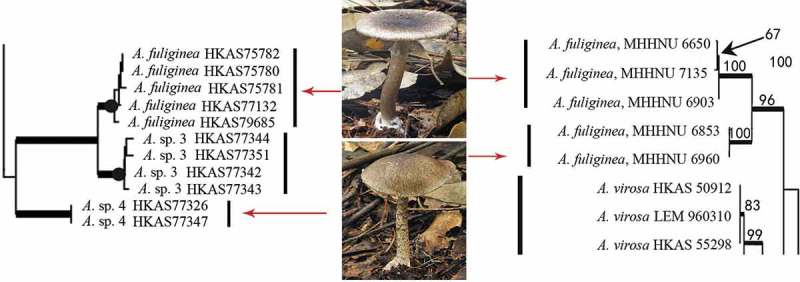
Two sub-clades of *A. fuliginea* in two phylogenetic trees (parcel) of *Amanita* based on ITS sequences (left: Cai et al. 2014; right: Zhang et al. 2010). *Amanita* sp. 4 in left tree is corresponding to *A. fuliginea* MHHNU 6853 and 6960 in right tree.

Many *Amanita* species contain one or more varieties or forma (Tulloss et al. 1995; Yang 2005). How to definite these infraspecies-level taxa remains a challenge. For some saprophytic basidiomycetes such as *Flammulina* and *Oudemansiella*, mating compatibility test is often used (Petersen and Halling 1993; Petersen et al. 1999). Unfortunately, most amanitas are EM and difficult to culture in the laboratory. Thus, mating test is unsuitable to identify their inter-fertility, so as to assign varieties and forma within *Amanita* species. Instead, the genealogical concordance phylogenetic analysis based on DNA nucleotide sequences has become popular in species and infraspecies recognition. According to the internal transcribed spacer (ITS) sequences analyses, Zhang et al. (2004) found four samples of *Amanitaparvipantherina* from different geographical localities and with different colours and morphologies in their fruit bodies all belonged to the same species. Based on multilocus DNA sequence data, Geml et al. (2008) confirmed the existence of several distinct phylogenetic species within *Amanitamuscaria*. Zhang et al. (2010) found two sub-clades within *Amanitafuliginea* and suggested that they should be named different forma or even different species. Indeed, recently, Cai et al. (2014) confirmed that these two sub-clades represented two different species (Figure 2).

**Figure 3.**
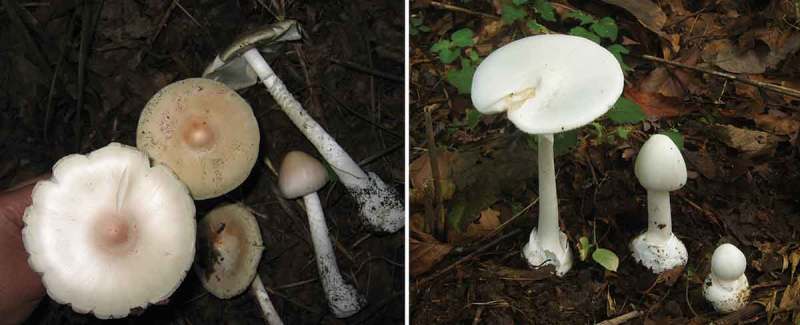
A ‘normal’ form (left) and an alba form (right) of *A. pallidorosea*.

Albefaction is a common phenomenon in *Amanita* species. Here, albefaction refers to white varieties, forma or morphotypes in some coloured *Amanita* species. Indeed, ‘var. alba’ or ‘f. alba’ has been reported in many species of this genus (Tulloss et al. 1995; Yang 2005). A putative reason for albefaction is mutation in genes related to pigment synthesis, though the specific mechanisms and process are not clear. For some species, e.g. *Amanitasubjunquillea*, albefaction is accompanied by other genetic changes. However, for other species, e.g. *Amanitapallidorosea*, the white morphotypes showed no obvious change except fruiting body colour, with natural fruiting bodies forming a continuous redistribution of colours and morphotypes (Zhang et al. 2010) (Figure 3).

**Figure 4.**
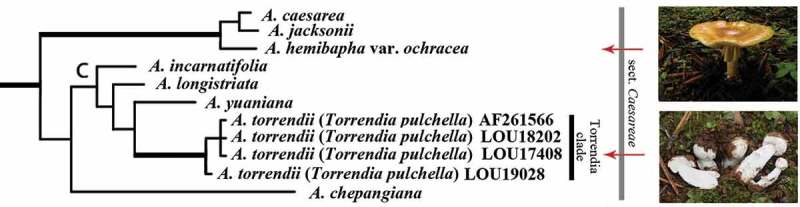
*Torrendia* and *Amanita* in a phylogenetic tree based on nLSU sequences (Justo et al. 2010).

### Gasteromycetation

Gasteromycetation has happened independently several times in different groups of fungi (Hibbett 2007). Secotioid and gasteroid forms also occur in genus *Amanita* as well as in some other groups of Basidiomyceta (Yang 2011). A secotioid genus *Torrendia* and a gasteroid genus *Amarrendia* Bougher & T. Lebel were postulated as close relatives of agaricoid amanitas over 60 years ago (Malencon 1955; Bas 1975). These hypotheses were later confirmed by molecular sequence information (Moncalvo et al. 2002). In 2010, Justo et al. (2010) formally transferred members of *Torrendia* and *Amarrendia* to genus *Amanita*. In addition, they suggested that the Mediterranean climate was responsible for the convergent evolution of these sequentrate fungi (Figure 4).

## Phylogeography

### Distribution patterns

Studies on geographic distribution patterns are fundamental for understanding the phylogeographic history of all organisms. We note that due to recent taxonomic revisions early literature on the distribution of some *Amanita* species may be outdated. One example is *Amanitagemmata*, a species originally described from Europe (Fries 1838) and later reported from North America (Coker 1917; Jenkins 1986) and eastern Asia (Nagasawa and Hongo 1985). Later molecular phylogenetic analysis (Zhang et al. 2004) showed that the so-called *A. gemmata* in North America and eastern Asia actually belong to species distinctly different from *A. gemmata* in Europe. These and other analyses suggest that *Amanita* species are more endemic than previously thought. For example, *Amanitaexitialis* is restricted to South China and southwestern China, *A. fuliginea* in tropical and subtropical East Asia, and *Amanitavirosa* in Europe and northeast Asia (Cai et al. 2014). However, there are several widely distributed species. *A. muscaria*, the type species of genus *Amanita*, is found in Europe (Moser 1983), North America (Jenkins 1986) and temperate eastern Asia (Imazeki and Hongo 1987). Oda et al. (2004) analysed the biogeography of *A. muscaria* based on ITS and β-tubulin sequences, separating it into at least three groups (Eurasian, Eurasian subalpine and North American). Geml et al. (2006, 2008) drew a similar conclusion about the phylogeographic structure and suggested that *A. muscaria* likely originated from the Siberian-Beringian region. *Amanitapantherina* is another widespread species found in Europe (Gilbert 1941), Asia (Imazeki and Hongo 1987), Africa (Reid and Eicker 1991), and North and Central America (Tulloss et al. 1995). This species is divided into at least two groups, the North American group and the Eurasian group. The relationships among samples from within both Eurasia and North America were closer to each other than the relationships among samples from between the two continents (Oda et al. 2004).

### Dispersal

Due to the lack of fossil records, the place and time for the origination of the genus *Amanita* are still uncertain. Current evidence suggests that members of this genus were present before the break-up of Gondwana and hence geographical populations have likely been isolated since then through continental drift (Cai et al. 2014). If this were the case, we should find endemic amanitas from the southern hemisphere. The results of investigation in South America (Bas 1978; Garrido and Bresinsky 1985; Bas and de Meijer 1993) were consistent with this hypothesis. However, long-distance migration is also possible. A study based on phylogenetic analysis and ancestral area reconstructions suggested that lethal amanitas (Section *Palloideae*) probably originated in the palaeotropical zone in the Palaeocene, migrated from the Eurasian continent to North America through the Beringian Land Bridge, and then extended to Central America during Oligocene to Miocene (Cai et al. 2014). Similarly, a recent study on edible amanitas (Section *Caesareae*) indicated that this group probably originated between the Palaeocene and Eocene in a Palaeotropical setting, most likely in Africa, subsequently dispersed into other temperate and tropical areas during the Miocene and Pliocene (Sánchez-Ramírez et al. 2015). The results of these studies are in agreement with the Eurasia-North America disjunct distribution pattern or the Eurasia-North/Central America distribution pattern for some species or sister species in this genus.

While oceans are important barriers restricting the dispersal of *Amanita* species, other factors such as deserts and mountains may also play a role similar to that of ocean in terms of vicariance. Tulloss (2005) found that Arizona in southwestern US shared few *Amanita* species with New Jersey and Long Island regions in northeastern US. However, southwestern US shared many species with Central and South America as far as Colombia. Since most *Amanita* species are EM fungi, their dispersals were likely accompanied by the dispersals of host plants. For example, the border of the Andean Colombian region appears to be the ‘end of the line’ for amanitas associated with *Quercus* and members of the Pinaceae (Tulloss 2005). This region is also the ‘end of the line’ for trees in the *Quercus* genus and several Pinaceae genera (Manos and Stanford 2001; Lin et al. 2010). Many amanitas from the south or east of this region are symbionts of leguminous or polygonaceous plants (Bas 1978). Whether these amanitas were associated with their current host plants from the initial stage or switched from other plants remains uncertain. As in many groups of Basidiomycetes, basidiospores likely play important roles in the dispersal of *Amanita* species. Theoretically, basidiospores may disperse by air flow for thousands of kilometres. However, a recent study found that most basidiospores of amanitas could only disperse for very limited distance. Li (2005) studied the release and dispersal of basidiospores from *A. muscaria* var. *alba*, and found that fewer than 2% of basidiospores dispersed to areas beyond 5.2 m from the basidiomata. Although long-distance dispersal events are rare, migration via spores is more likely to explain the Eurasia-North America disjunct distribution pattern in some species (Geml et al. 2006, 2008).

### Effects of human activities

With the rapid developments of human societies and modern technologies, intercontinental travel and exchanges of goods have become more and more frequent. Some EM fungi including *Amanita* species have likely dispersed among continents with their host plants due to human activities. For example, *A. phalloides*, a notoriously poisonous mushroom originally described from Europe (Fries 1821) and repeatedly recorded in North America from the nineteenth century (Schweinitz 1834; Harknes and Moore 1880; Taylor 1897), was considered an introduced fungus in North America (Pringle and Vellinga 2006). A subsequent phylogeographical analysis based on six loci supported this hypothesis (Pringle et al. 2009), which was further confirmed by Wolfe et al. (2010). In addition, *A. phalloides* is known to have been artificially introduced to Australia, New Zealand and South Africa together with its host plants (Dunstan et al. 1998). *A. muscaria* is another EM fungus known to be introduced to Australia (Sawyer et al. 2001). Thus, human activity is a major factor that needs to be considered in the phylogeographical researches of *Amanita* mushrooms.

## Population genetics

One of the fundamental properties of fungal populations in nature is genet size. A genet refers to a group of sporocarps that have identical genetic backgrounds and resulted from the same mating event (Zhou et al. 2000, 2001). Genet size differs among EM fungal species, ranging from a few metres to 100 m in diameter (Dahlberg 2001; Sawyer et al. 2001). Molecular methods provide more sensitive and effective markers in the identification of genet of EM fungi and are now being widely applied in population genetic studies of *Amanita*, as well as in other group of fungi (e.g. Timonen et al. 1997; Bonello et al. 1998; Junghans et al. 1998; Sawyer et al. 1999). Polymerase chain reaction-restriction fragment length polymorphism, random amplified polymorphic DNA, amplified fragment length polymorphism (AFLP), inter-simple sequence repeat (ISSR) and single nucleotide polymorphisms (SNPs) are popular polymorphic markers applied in population genetics of EM fungi. Among them, AFLP and ISSR markers are the most widely used in population genetics of *Amanita* species. High-throughput SNP is another recent type of molecular marker. It has the advantages of high stability, low mutation rate, co-dominance and ease of scoring. However, SNPs have not been used in population genetic studies of *Amanita* mushrooms.

Our knowledge of population genetics of genus *Amanita* is currently limited to only a few taxa. Redecker et al. (2001) determined the size of genets of three EM fungi in field sites in coastal northern California using AFLPs fingerprinting. The results showed *Amanitafranchetii* formed small genets with the biggest at 4.7 m across. Sawyer et al. (2003) studied the distribution and persistence of *A. muscaria* genotypes in three *Pinus radiata* plantations in New South Wales, Australia. The presence of common genotypes at the three sites indicated that they were introduced as vegetative inocula when seedlings were planted and have persisted for up to 36 years. Population structure and spreading strategy of a species in natural forests is different from that in plantation forests. Genotypes of five Australian *Amanita* species, *Amanitaalboverrucosa*, *Amanitaochrophylla*, *Amanitapyramidifera*, *Amanitaconicoverrucosa* and *Amanitapunctata*, were investigated using ISSR fingerprinting (Sawyer et al. 2003). Genotypes of *A. ochrophylla*, *A. conicoverrucosa* and *A. punctata* were spread over areas with the largest dimensions ranging from 10 to 60 m, suggesting evidence of vegetative spread via large below-ground mycelial genets. In contrast, genotypes of *A. alboverrucosa* were more spatially restricted, suggesting recent establishment via basidiospores and more limited below-ground vegetative spread. Interestingly, two groups of *A. pyramidifera* basidiomes with the same genotype were separated by 600 m, suggesting the vegetative tissue might have been moved by vehicular activity. The population genetic structure of *Amanitamanginiana* in a natural forest in southwest China was examined over two years using ISSR markers (Liang et al. 2005). In contrast to the relatively large genets, the results indicated that each sporocarp represented a single genet, and no identical genets were found between 2001 and 2002. Although the genetic variances were mainly found among individuals of the same year, the variance between years was statistically significant.

## Prospects

Extensive collection, precise identification and comprehensive evaluations and comparisons are fundamental issues of taxonomic studies. Further, taxonomy is the foundation for phylogeographic investigations. In some areas such as Europe and North America, fungal taxonomy studies have been carried out for about two centuries. In contrast, surveys of fungal flora are still in their preliminary stages in tropical Africa and South America. It is anticipated that new taxa of *Amanita* will be discovered in these regions in the future, and these taxa will contribute to a better understanding of the origin and evolution history of this genus. Molecular-data-based systematics and taxonomies have evolved very rapidly and revealed a large number of cryptic species. Documentation and integration of these cryptic species into the established framework are urgent tasks in the near future. Interestingly, even in geographic areas that have been intensively studied by taxonomists, new taxa continue to emerge. Recent studies have shown that many morphological species such as *Amanitapseudoporphyria*, *Amanitavaginata* and *Amanita hemibapha* are actually species complexes with each containing multiple divergent lineages. More extensive molecular phylogenetic studies using sequences at multiple loci should help reveal the cryptic species within each of these species complexes (Yang 2005).

Recently, molecular phylogenetic analyses of a few selected groups of *Amanita* have helped reveal their origins and evolution (Oda et al. 1999, 2004; Geml et al. 2006, 2008; Cai et al. 2014). It is hoped that future phylogeographic studies will provide a more comprehensive picture of the origin and evolution at the genus level.

Population genetic studies of *Amanita* are still at an early stage. Up to now, only in a few species have been analysed (Redecker et al. 2001; Sawyer et al. 2003; Liang et al. 2005). Both spatial and temporal factors need to be considered when analysing natural populations. Spatially, a diversity of scales, from fine local scale to regional-, national- and global-level investigations, is needed. To examine how fungal populations change over time, long-term monitoring is also needed. Since *Amanita* mushrooms include both lethal and gourmet species, studies on population genetics of these species will reveal the differences between poisonous and edible mushrooms on strategies of reproduction, dispersal and succession. The advent of molecular biology, decreasing cost of sequencing and increasing availability of sequenced genomes made it easier to exploit new markers (e.g. SNP markers) for fungal population genetic analyses. Population genetics will not only help us to understand these species better, but also benefit to forest management and conservation of some valued edible species (e.g. *A. hemibapha, A. caesarea*).

## Disclosure statement

No potential conflict of interest was reported by the authors.

## References

[CIT0001] Bas C. 1975 A comparison of *Torrendia* (Gasteromycetes) with *Amanita* (Agaricales). Beih Nov Hedwig. 51:53–61.

[CIT0002] Bas C 1978 Studies in *Amanita*-I. Some species from Amazonia. Persoonia. 10:1–22.

[CIT0003] Bas C 2000 A broader view on *Amanita*. Bollettino del Gruppo Micologico g Bresadola. 43:9–12.

[CIT0004] Bas C, de Meijer AAR 1993 *Amanita grallipes*, a new species in *Amanita* subsection *Vittadiniae* from southern Brazil. Persoonia. 15:345–350.

[CIT0005] Bhatt RP, Tulloss RE, Semwal KC, Bhatt VK, Moncalvo JM, Stephenson SL 2003 *Amanitaceae* from India. A critically annotated checklist. Mycotaxon. 88:249–270.

[CIT0006] Bonello P, Bruns TD, Gardes M 1998 Genetic structure of a natural population of the ectomycorrhizal fungus *Suillus pungens*. New Phytol. 138:533–542. doi:10.1046/j.1469-8137.1998.00122.x

[CIT0007] Cai Q, Tulloss RE, Tang LP, Tolgor B, Zhang P, Chen ZH, Yang ZL 2014 Multi-locus phylogeny of lethal amanitas: implications for species diversity and historical biogeography. BMC Evol Biol. 14:143–158. 24950598 10.1186/1471-2148-14-143 PMC4094918

[CIT0008] Coker WC 1917 The *Amanitas* of the eastern United States. J Elisha Mitchell Sci Soc. 33:1–88.

[CIT0009] Dahlberg A 2001 Community ecology of ectomycorrhizal fungi: an advancing interdisciplinary field. New Phytol. 150:555–562. doi:10.1046/j.1469-8137.2001.00142.x

[CIT0010] Deng W-Q, Li T-H, Li P, Yang ZL 2014 A new species of *Amanita* section *Lepidella* from South China. Mycol Prog. 13:211–217. doi:10.1007/s11557-013-0906-6

[CIT0011] Drehmel D, Moncalvo JM, Vilgalys R 1999 Molecular phylogeny of *Amanita* based on large-subunit ribosomal DNA sequences: implications for taxonomy and character evolution. Mycologia. 91:610–618.

[CIT0012] Dunstan WA, Dell B, Malajczuk N 1998 The diversity of ectomycorrhizal fungi associated with introduced *Pinus* spp. in the southern hemisphere, with particular reference to western Australia. Mycorrhiza. 8:71–79. doi:10.1007/s005720050215

[CIT0013] Eicker A, Van Greuning J, Reid D 1993 *Amanita reidii*: a new species from South Africa. Mycotaxon. 47:433–437.

[CIT0014] Fries EM 1821 Systema mycologicum I. Gryphiswaldiae: Ernesti Mauritii.

[CIT0015] Fries EM 1838 Epicrisis Systematis Mycologici. Uppsala (Sweden): Typographia Academica.

[CIT0016] Garrido N, Bresinsky A 1985 *Amanita merxmuelleri* (Agaricales), eine neue art aus *Nothofagus*-Wäldern Chiles. Bot Jahrb Syst. 107:521–540.

[CIT0017] Geml J, Laursen GA, O’neill K, Nusbaum HC, Taylor D 2006 Beringian origins and cryptic speciation events in the fly agaric (*Amanita muscaria*). Mol Ecol. 15:225–239. doi:10.1111/j.1365-294X.2005.02799.x 16367842

[CIT0018] Geml J, Tulloss RE, Laursen GA, Sazanova NA, Taylor DL 2008 Evidence for strong inter- and intracontinental phylogeographic structure in *Amanita muscaria*, a wind-dispersed ectomycorrhizal basidiomycete. Mol Phylogenet Evol. 48:694–701. doi:10.1016/j.ympev.2008.04.029 18547823

[CIT0019] Gilbert EJ 1941 Amanitaceae. Iconographia Mycologica. 27:201–427.

[CIT0020] Harkness HW, Moore JP 1880 Catalogue of the Pacific Coast Fungi. San Francisco (CA): California Academy of Sciences.

[CIT0021] Hibbett DS 2007 After the gold rush, or before the flood? Evolutionary morphology of mushroom-forming fungi (Agaricomycetes) in the early 21st century. Mycol Res. 111:1001–1018. doi:10.1016/j.mycres.2007.01.012 17964768

[CIT0022] Imazeki R, Hongo T 1987 Colored illustrations of mushrooms of Japan. Vol. 1 Osaka: Hoikusha.

[CIT0023] Jenkins DT 1986 *Amanita* of North America. Eureca (CA): Mad River Press.

[CIT0024] Junghans DT, Gomes EA, Guimarães WV, Barros EG, Araújo EF 1998 Genetic diversity of the ectomycorrhizal fungus *Pisolithus tinctorius* based on RAPD-PCR analysis. Mycorrhiza. 7:243–248. doi:10.1007/s005720050187 24578049

[CIT0025] Justo A, Morgenstern I, Hallen-Adams HE, Hibbett DS 2010 Convergent evolution of sequestrate forms in *Amanita* under Mediterranean climate conditions. Mycologia. 102:675–688. doi:10.3852/09-191 20524599

[CIT0026] Li D-W 2005 Release and dispersal of basidiospores from *Amanita muscaria* var. *alba* and their infiltration into a residence. Mycol Res. 109:1235–1242. doi:10.1017/S0953756205003953 16279416

[CIT0027] Li F, Cai Q 2014 *Amanita heishidingensis*, a new species of *Amanita* sect. *Lepidella* from China. Mycol Prog. 13:1191–1197. doi:10.1007/s11557-014-1008-9

[CIT0028] Liang Y, Guo L-D, Ma K-P 2005 Population genetic structure of an ectomycorrhizal fungus *Amanita manginiana* in a subtropical forest over two years. Mycorrhiza. 15:137–142. doi:10.1007/s00572-004-0311-8 15164273

[CIT0029] Lin C-P, Huang J-P, Wu C-S, Hsu C-Y, Chaw S-M 2010 Comparative chloroplast genomics reveals the evolution of Pinaceae genera and subfamilies. Genome Biol Evol. 2:504–517. doi:10.1093/gbe/evq036 20651328 PMC2997556

[CIT0030] Malencon G 1955 Le dévelopement de Torrendia pulchella Bres. et son importance morphogénétique. Rev Mycol. 20:81–130.

[CIT0031] Manos PS, Stanford AM 2001 The historical biogeography of Fagaceae: tracking the tertiary history of temperate and subtropical forests of the Northern Hemisphere. Int J Plant Sci. 162:77–93. doi:10.1086/323280

[CIT0032] Moncalvo J-M, Vilgalys R, Redhead SA, Johnson JE, James TY, Aime MC, Hofstetter V, Verduin SJW, Larsson E, Baroni TJ, et al. 2002 One hundred seventeen clades of euagarics. Mol Phylogenet Evol. 23:357–400. doi:10.1016/S1055-7903(02)00027-1 12099793

[CIT0033] Moser M 1983 Keys to agarics and boleti (polyporales, boletales, agaricales, russulales). London: Roger Phillips.

[CIT0034] Nagasawa E, Hongo T 1985 Some agarics from the San-in district, Japan. Memoirs of National Science Museum, Tokyo. 18:73–88.

[CIT0035] Nagasawa E, Mitani S 2000 A new species of *Amanita* section *Lepidella* from Japan. Memoirs of the National Science Museum, Tokyo. 32:93–97.

[CIT0036] Neville P, Poumarat S 2004 Amaniteae: *Amanita, Limacella* & *Torrendia*. Fungi Europaei Vol. 9 Alssio: Edizioni Candusso.

[CIT0037] Oda T, Tanaka C, Tsuda M 1999 Molecular phylogeny of Japanese *Amanita* species based on nucleotide sequences of the internal transcribed spacer region of nuclear ribosomal DNA. Mycoscience. 40:57–64. doi:10.1007/BF02465674

[CIT0038] Oda T, Tanaka C, Tsuda M 2001 *Amanita imazekii*-a new species in *Amanita* section *Caesareae*. Mycologia. 93:1231–1234.

[CIT0039] Oda T, Tanaka C, Tsuda M 2002a Two new species of *Amanita* from Japan. Mycoscience. 43:351–355. doi:10.1007/S102670200051

[CIT0040] Oda T, Tanaka C, Tsuda M 2002b *Amanita concentrica*: a new species in *Amanita* section *Amanita* from Japan. Mycoscience. 43:81–83. doi:10.1007/s102670200013

[CIT0041] Oda T, Tanaka C, Tsuda M 2004 Molecular phylogeny and biogeography of the widely distributed *Amanita* species, A. muscaria and A. pant henna. Mycol Res. 108:885–896. doi:10.1017/S0953756204000620 15449593

[CIT0042] Oda T, Yamazaki T, Tanaka C, Terashita T, Taniguchi N, Tsuda M 2002c *Amanita ibotengutake* sp. nov., a poisonous fungus from Japan. Mycol Prog. 1:355–365. doi:10.1007/s11557-006-0032-9

[CIT0043] Persoon CH 1801 Synopsis methodica fungorum. Gottingae: H Dieterich.

[CIT0044] Petersen RH, Halling RE 1993 Mating systems in the Xerulaceae: *Oudemansiella*. Trans Mycol Soc Jpn. 34:409–422.

[CIT0045] Petersen RH, Hughes KW, Redhead SA, Psurtseva N, Methven AS 1999 Mating systems in the *Xerulaceae* (*Agaricales, Basidiomycotina*): *Flammulina*. Mycoscience. 40:411–426. doi:10.1007/BF02464396

[CIT0046] Pringle A, Adams RI, Cross HB, Bruns TD 2009 The ectomycorrhizal fungus *Amanita phalloides* was introduced and is expanding its range on the west coast of North America. Mol Ecol. 18:817–833. doi:10.1111/j.1365-294X.2008.04030.x 19207260

[CIT0047] Pringle A, Vellinga EC 2006 Last chance to know? using literature to explore the biogeography and invasion biology of the death cap mushroom *Amanita phalloides* (Vaill. Ex fr.: Fr.) link. Biol Invasions. 8:1131–1144. doi:10.1007/s10530-005-3804-2

[CIT0048] Redecker D, Szaro TM, Bowman RJ, Bruns TD 2001 Small genets of *Lactarius xanthogalactus, Russula cremoricolor* and *Amanita francheti* in late-stage ectomycorrhizal successions. Mol Ecol. 10:1025–1034. doi:10.1046/j.1365-294X.2001.01230.x 11348508

[CIT0049] Reid DA, Eicker A 1991 South African fungi: the genus *Amanita*. Mycol Res. 95:80–95. doi:10.1016/S0953-7562(09)81364-6

[CIT0050] Sánchez-Ramírez S, Tulloss RE, Amalfi M, Moncalvo J-M 2015 Palaeotropical origins, boreotropical distribution and increased rates of diversiﬁcation in a clade of edible ectomycorrhizal mushrooms (*Amanita* section *Caesareae*). J Biogeogr. 42:351–363. doi:10.1111/jbi.12402

[CIT0051] Sawyer NA, Chambers SM, Cairney JWG 1999 Molecular investigation of genet distribution and genetic variation of *Cortinarius rotundisporus* in eastern Australian sclerophyll forests. New Phytol. 142:561–568. doi:10.1046/j.1469-8137.1999.00417.x

[CIT0052] Sawyer NA, Chambers SM, Cairney JWG 2001 Distribution and persistence of *Amanita muscaria* genotypes in Australian *Pinus radiata* plantations. Mycol Res. 105:966–970. doi:10.1017/S0953756201004488

[CIT0053] Sawyer NA, Chambers SM, Cairney JWG 2003 Distribution of *Amanita* spp. genotypes under eastern Australian sclerophyll vegetation. Mycol Res. 107:1157–1162. doi:10.1017/S0953756203008426 14635764

[CIT0054] Schweinitz LD 1834 Synopsis fungorum in America boreali. Trans Am Philosophical Soc. 4:141–316.

[CIT0055] Simmons C, Henkel T, Bas C 2002 The genus *Amanita* in the Pakaraima mountains of Guyana. Persoonia. 17:563–582.

[CIT0056] Taylor T 1897 Student’s hand-book of mushrooms of America Edible and Poisonous. Washington, DC: A.R. Taylor.

[CIT0057] Timonen S, Tammi H, Sen R 1997 Outcome of interactions between genets of two *Suillus* spp. and different Pinus sylvestris genotype combinations: identity and distribution of ectomycorrhizas and effects on early seedling growth in N-limited nursery soil. New Phytol. 137:691–702. doi:10.1046/j.1469-8137.1997.00871.x

[CIT0058] Tulloss RE 2005 *Amanita*–distribution in the Americas, with comparison to eastern and southern Asia and notes on spore character variation with latitude and ecology. Mycotaxon. 93:189–231.

[CIT0059] Tulloss RE, Bhatt R, Stephenson S, Kumar A 1995 Studies on *Amanita* (*Amanitaceae*) in West Virginia and adjacent areas of the Mid-Appalachians, preliminary results. Mycotaxon. 56:243–293.

[CIT0060] Tulloss RE, Iqbal SH, Khalid AN, Bhatt RP, Bhatt VK 2001 Studies in *Amanita* (*Amanitaceae*) from southern Asia. I. some species of Pakistan’s Northwest Frontier Province. Mycotaxon. 77:455–490.

[CIT0061] Tulloss RE, Lindgren JE 1994 *Amanita novinupta—*a rubescent, white species from the western United States and southwestern Canada. Mycotaxon. 51:179–190.

[CIT0062] Tulloss RE, Ovrebo CL, Halling RE 1992 Studies on *Amanita* (Agaricales) from Andean Colombia. Mem New York Bot Gard. 66:1–46.

[CIT0063] Weiß M, Yang ZL, Oberwinkler F 1998 Molecular phylogenetic studies in the genus *Amanita*. Can J Bot. 76:1170–1179.

[CIT0064] Wolfe BE, Richard F, Cross HB, Pringle A 2010 Distribution and abundance of the introduced ectomycorrhizal fungus *Amanita phalloides* in North America. New Phytol. 185:803–816. doi:10.1111/j.1469-8137.2009.03097.x 20002314

[CIT0065] Wood AE 1997 Studies in the genus *Amanita* (Agaricales) in Australia. Austral Syst Bot. 10:723–854.

[CIT0066] Yang ZL 1997 Die *Amanita*-Arten von Suedwestchina. Bibl Mycol. 170:1–240.

[CIT0067] Yang ZL 2000a Species diversity of the genus *Amanita* (Basidiomycetes) in China. Acta Botanica Yunnanica. 22:135–142.

[CIT0068] Yang ZL 2000b On taxonomic studies of the Chinese *Amanitae*. Mycosystema. 19:435–440.

[CIT0069] Yang ZL 2002 Revision of *Amanita* collections made from Jilin Province, Northeastern China. Mycotaxon. 83:67–76.

[CIT0070] Yang ZL 2005 Flora fungorum sinicorum. Vol. 27 Amanitaceae(in Chinese) Beijing: Science Press.

[CIT0071] Yang ZL 2011 Molecular techniques revolutionize knowledge of basidiomycete evolution. Fungal Divers. 50:47–58. doi:10.1007/s13225-011-0121-1

[CIT0072] Yang ZL, Doi Y 1999 A contribution to the knowledge of *Amanita* (Amanitaceae, Agaricales) in Japan. Bull Natl Sci Museum Ser B. 25:107–130.

[CIT0073] Yang ZL, Li TH 2001 Notes on three white *Amanitae* of section *Phalloideae* (Amanitaceae) from China. Mycotaxon. 78:439–448.

[CIT0074] Yang ZL, Li TH, Wu XL 2001 Revision of *Amanita* collections made from Hainan, southern China. Fungal Divers. 6:146–165.

[CIT0075] Yang ZL, Oberwinkler F 1999 Die Fruchtköperentwicklung von *Amanita* muscaria (Basidiomycetes). Nova Hedwigia. 68:441–468.

[CIT0076] Yang ZL, Weiß M, Kottke I, Oberwinkler F, Nehls U, Guttenberger M, Hampp R 1999 Chapter 8. *Amanita* In: Cairney JWG, Chambers SM, editors Ectomycorrhizal Fungi: key Genera in profile. Germany: Springer-Verlag; p. 201–230.

[CIT0077] Yang ZL, Weiß M, Oberwinkler F 2004 New species of *Amanita* from eastern Himalayas and adjacent regions. Mycologia. 96:636–646. 21148883

[CIT0078] Yang ZL, Zhang LF 2002 Revision of collections of *Amanita* (Agaricales) from Hunan Province, central China. Acta Botanica Yunnanica. 24:715–722.

[CIT0079] Zhang LF, Yang JB, Yang ZL 2004 Molecular phylogeny of eastern Asian species of *Amanita* (Agaricales, Basidiomycota): taxonomic and biogeographic implications. Fungal Divers. 17:219–238.

[CIT0080] Zhang P, Chen ZH, Xiao B, Tolgor B, Bao HY, Yang ZL 2010 Lethal amanitas of East Asia characterized by morphological and molecular data. Fungal Divers. 42:119–133. doi:10.1007/s13225-010-0018-4

[CIT0081] Zhou ZH, Miwa M, Hogetsu T 2000 Genet distribution of ectomycorrhizal fungus *Suillus grevillei* populations in two Larix kaempferi stands over two years. J Plant Res. 113:365–374. doi:10.1007/PL00013944

[CIT0082] Zhou ZH, Miwa M, Matsuda Y, Hogetsu T 2001 Spatial distribution of the subterranean mycelia and ectomycorrhizae of *Suillus grevillei* genets. J Plant Res. 114:179–185. doi:10.1007/PL00013981

